# A New Statistic to Evaluate Imputation Reliability

**DOI:** 10.1371/journal.pone.0009697

**Published:** 2010-03-15

**Authors:** Peng Lin, Sarah M. Hartz, Zhehao Zhang, Scott F. Saccone, Jia Wang, Jay A. Tischfield, Howard J. Edenberg, John R. Kramer, Alison M.Goate, Laura J. Bierut, John P. Rice, for the COGA Collaborators COGEND Collaborators, GENEVA

**Affiliations:** 1 Department of Psychiatry, Washington University, St. Louis, Missouri, United States of America; 2 Department of Genetics, Rutgers University, Piscataway, New Jersey, United States of America; 3 Department of Biochemistry and Molecular Biology, Indiana University School of Medicine, Indianapolis, Indiana, United States of America; 4 Department of Psychiatry, University of Iowa School of Medicine, Iowa City, Iowa, United States of America; Universite de Montreal, Canada

## Abstract

**Background:**

As the amount of data from genome wide association studies grows dramatically, many interesting scientific questions require imputation to combine or expand datasets. However, there are two situations for which imputation has been problematic: (1) polymorphisms with low minor allele frequency (MAF), and (2) datasets where subjects are genotyped on different platforms. Traditional measures of imputation cannot effectively address these problems.

**Methodology/Principal Findings:**

We introduce a new statistic, the imputation quality score (IQS). In order to differentiate between well-imputed and poorly-imputed single nucleotide polymorphisms (SNPs), IQS adjusts the concordance between imputed and genotyped SNPs for chance. We first evaluated IQS in relation to minor allele frequency. Using a sample of subjects genotyped on the Illumina 1 M array, we extracted those SNPs that were also on the Illumina 550 K array and imputed them to the full set of the 1 M SNPs. As expected, the average IQS value drops dramatically with a decrease in minor allele frequency, indicating that IQS appropriately adjusts for minor allele frequency. We then evaluated whether IQS can filter poorly-imputed SNPs in situations where cases and controls are genotyped on different platforms. Randomly dividing the data into “cases” and “controls”, we extracted the Illumina 550 K SNPs from the cases and imputed the remaining Illumina 1 M SNPs. The initial Q-Q plot for the test of association between cases and controls was grossly distorted (λ = 1.15) and had 4016 false positives, reflecting imputation error. After filtering out SNPs with IQS<0.9, the Q-Q plot was acceptable and there were no longer false positives. We then evaluated the robustness of IQS computed independently on the two halves of the data. In both European Americans and African Americans the correlation was >0.99 demonstrating that a database of IQS values from common imputations could be used as an effective filter to combine data genotyped on different platforms.

**Conclusions/Significance:**

IQS effectively differentiates well-imputed and poorly-imputed SNPs. It is particularly useful for SNPs with low minor allele frequency and when datasets are genotyped on different platforms.

## Introduction

Genome-wide association studies (GWAS) represent a powerful approach to the identification of genetic variants involved in common human diseases[1]. GWAS use commercial SNP microarrays to genotype large numbers of genetic markers. However, SNP microarrays currently can only genotype up to one million of the 9–10 million common SNPs in the assembled human genome [2]. In addition, for a typical case-control design, several thousand cases and several thousand controls may be needed for adequate power to detect associations[3]. With little cost, imputation can boost power both by increasing SNP coverage and by combining samples from similar studies. Based on haplotypes from the International HapMap project[4], imputation infers untyped variants from known genotypes. The inference uses one of several model-based methods, and the resulting imputed SNPs can be tested for association with a phenotype [5]. The power of this method has been demonstrated in the literature where several groups have found novel causal genes [6], [7], [8], [9].

There are two situations where researchers avoid imputation due to increased error in imputation: (1) SNPs with minor allele frequency less than 1% [1], [6], [10], and (2) association studies where cases and controls are genotyped on different platforms. Imputation accuracy, calculated for each SNP as the proportion of genotypes correctly classified, is the gold standard for evaluating the quality of imputation. Unfortunately, it is an inadequate filter in both of these circumstances. For the majority of SNPs, imputation programs such as IMPUTE [5], MACH[10], and BEAGLE[11], have very high imputation accuracy [5], [11], [12], [13]. However, the use of imputation accuracy in low frequency SNPs to evaluate imputation quality can be misleading. When the minor allele frequency of a SNP is less than 5%, a program could randomly assign the two alleles to the sample only using the minor allele frequency and achieve more than 90% accuracy. Although SNPs with low minor allele frequencies (MAF<5%) are referred to as uncommon SNPs, they represent more than 30% of SNPs in the HapMap Phase II CEU population, and this proportion is even higher in African populations[2]. This problem assessing imputation accuracy in lower frequency SNPs means that a large part of the genome will not be adequately interrogated using imputation.

The second problematic situation for imputation is where cases and controls are genotyped on different platforms. This is problematic because imputation error can vary between cases and controls, causing increased rates of false positives in association studies. There is no known method for effectively filtering the poorly imputed SNPs from the well imputed SNPs on different platforms. Although this situation has been avoided by researchers, it is an important application. Large studies such as Wellcome Trust and the NIMH GAIN samples use common controls that could be used in other studies to gain power [1], [14]. But, if the primary datasets were genotyped on a different platform, imputation is necessary.

In order to assess the reliability of imputation with an emphasis on the less common SNPs and an interest in evaluating data imputed from different platforms, we introduce a new statistic, the imputation quality score (IQS). Partly motivated by Cohen's statistic *Kappa* to quantify rater agreement[15], IQS takes chance agreement into account and thus controls for allele frequencies. In this paper, we introduce IQS, demonstrate its value in situations of low minor allele frequencies, and demonstrate how it can be used to improve the type I error rate when cases and controls are genotyped on different platforms.

## Materials and Methods

### Ethics statement

De-identified data from the Study of Addiction: Genetics and Environment (SAGE) were analyzed for the research reported in this manuscript. SAGE consists of existing data from three genetic studies of addiction: the Collaborative Study on the Genetics of Alcoholism (COGA), the Collaborative Genetic Study of Nicotine Dependence (COGEND), and the Family Study of Cocaine Dependence (FSCD). All participants in COGA, COGEND and FSCD provided written informed consent for genetic studies and agreed to share their DNA and phenotypic information for research purposes. The institutional review boards at all data collection sites granted approval for data collected from COGA, COGEND and FSCD to be used for the Study of Addiction: Genetics and Environment. Specifically, approval was obtained from the Washington University Human Research Protection Office (for COGA, COGEND and FSCD), the State University of New York Downstate Medical Center Institutional Review Board (COGA), the University of Connecticut Health Center Human Subjects Protection Office (COGA), the Indiana University Research Compliance Administration (COGA), the University of California, San Diego Human Research Protections Program (COGA), the Howard University Institutional Review Board (COGA), The University of Iowa Human Subjects Office (COGA), and the Henry Ford Health System Institutional Review Board (COGEND). The second dataset was obtained from the National Institute of Mental Health Center for Collaborative Genetic Studies on Mental Disorders (http://www.nimhgenetics.org/) and was also de-identified.

### Methods

The computation of IQS requires the posterior probabilities of AA, AB and BB as output by the imputation program. For one SNP genotyped on N individuals, the probabilities can be readily tabulated into a 3×3 table where each cell, *n_ij_*, represents the number of individuals with true genotype *i* and imputed genotype *j* (Table 1). Note, in this scenario, *n_ij_* may not be an integer due to imputation probabilities being reported rather than imputed genotypes.

**Table 1:** Marginal cross classification of the genotypes used for the computation of IQS.

	True genotypes			
Imputed Genotypes	AA	AB	BB	Total
AA	*n_11_*	*n_12_*	*n_13_*	*n_1._*
AB	*n_21_*	*n_22_*	*n_23_*	*n_2._*
BB	*n_31_*	*n_32_*	*n_33_*	*n_3._*
Total	*n_.1_*	*n_.2_*	*n_.3_*	*n..*

IQS adjusts for minor allele frequency by comparing observed frequencies to expected frequencies.

We define the observed proportion of agreement (*P_o_*) as: 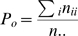



The observed proportion of agreement can be used to evaluate imputation reliability. But, like imputation accuracy and average maximum posterior probability, it can overestimate reliability for uncommon SNPs because it is not adjusted for “chance” agreement.

IQS adjusts for allele frequency by subtracting “chance” agreement from the “observed” agreement. Similar to *P_o_*, “chance” agreement (*P_c_*) is computed as the sum of the products of marginal frequencies that would occur if genotypes are called at random using the same marginal rates: 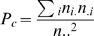



IQS is then computed by subtracting the chance agreement from the observed agreement and dividing by the maximum possible value of the numerator. The value of one indicates a perfect match, and negative values indicate that the imputation program performed worse than chance. 
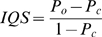



In addition, the calculation of IQS can be expanded to evaluate non-random error. When cases and controls are genotyped on different platforms (e.g., cases genotyped on the Affymetrix array and controls genotyped on the Illumina array), some SNPs are not genotyped in either array but are imputed from their respective arrays. This imposes non-random errors on the imputed genotypes. In particular, if we combine these imputed genotypes together, it will inflate false positive rates. IQS can take this into account by incorporating marginal frequencies into the calculation. For instance, if imputation from the Illumina array reports that for a particular SNP, the probabilities of AA, AB and BB are *a_1_, a_2_, a_3_*, and imputation from the Affymetrix array reports that the probabilities for the three genotypes are *b_1_, b_2_, b_3_*, then *n_ij_* in the calculation of *P_o_* becomes
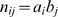



In this scenario, IQS provides a useful criterion to exclude unacceptable SNPs imputed from different sources.

### Data and imputation

The first dataset was collected as part of SAGE, one study in the Gene Environment Association (GENEVA) project (http://genevastudy.org/). Samples were genotyped on the Illumina Human 1 M array at the Center for Inherited Disease Research (CIDR) at Johns Hopkins University. The Illumina 1 M array has a total of 1,049,008 probes as SNP assays. All SNPs with a genotype call rate <98% were removed, as were SNPs with a Hardy-Weinberg exact p value <1×10^−4^. Additional data cleaning procedures were applied to ensure the highest possible data quality, including using HapMap controls, detection of gender and chromosomal anomalies, hidden relatedness, population structure, batch effects, Mendelian error detection, and duplication error detection[16]. The composition of the remaining project samples in terms of self-identified ethnicity is 2597 European Americans and 1264 African Americans, confirmed by principal component analysis. Among the 1,049,008 SNPs, 948,658 SNPs (90%) passed data cleaning procedures.

The second dataset consists of controls from the National Institute of Mental Health Center for Collaborative Genetic Studies on Mental Disorders (http://www.nimhgenetics.org/). A total of 418 subjects (controls) were genotyped using both the Affymetrix GeneChip Mapping 500 K Array Set and the Illumina HumanHap 550 K Array set and passed all cleaning procedures. All individuals in this study were European Americans with no evidence of heterogeneity, verified by principal component analysis[17]. All SNPs with a genotype call rate <95% were removed, as well as SNPs with a Hardy-Weinberg exact p value <1×10^−5^. After quality control, 447,250 autosomal SNPs were retained from the Affymetrix 500 K array, and 527,095 autosomal SNPs were retained from the Illumina 550 K array.

Imputation from each array to Hapmap SNPs was done by the program IMPUTE (https://mathgen.stats.ox.ac.uk/impute/impute_v0.5.html) [5]. European Americans were imputed using the CEU reference panel (HapMap release 22 - NCBI Build 36 dbSNP b126). African Americans were imputed separately using the YRI reference panel (HapMap release 22 - NCBI Build 36 dbSNP b126). We omitted sex chromosomes in this study because of the complication of imputation on these chromosomes. The Illumina 1 M array contains a small number of strand-ambiguous A/T C/G SNPs. Although Illumina provides strand information about those SNPs, we still found a few inconsistencies compared with the reference panel. In order to make sure that all SNPs were reported on the same strand, all strand-ambiguous A/T and C/G SNPs (5583 in total, 0.5% of all Illumina 1 M SNPs) were excluded from the comparison.

Imputation efficiency is calculated as the proportion of genotypes that had a maximum posterior probability greater than 0.9, as recommended by IMPUTE.

Association tests were done by the program SNPTEST with the “-proper” option[5]. With this option, SNPTEST runs a logistic regression based on the probability of genotype rather than dichotomous genotype, allowing the uncertainty of the imputation to be factored into the consideration [18].

### Statistical estimates of imputation quality

Both IQS and imputation accuracy compare true genotypes to imputed genotypes. Given that imputation is designed to infer unknown genotypes, one purpose of this paper was to use IQS to evaluate statistics that measure the quality of imputation *without* knowing the true genotype. The two statistics most commonly used for this purpose are the variance ratio (rsq_hat in MACH)[10] and the imputed information score (PROPER_INFO in SNPTEST) [5]. The variance ratio for a particular SNP is a ratio of the empirically observed variance (based on the imputation) to the expected binomial variance p(1-p), where p is the minor allele frequency[18]. As the amount of information available to impute the SNP decreases, the empirically observed variance decreases and the variance ratio approaches zero. The product of the variance ratio and sample size defines the ‘effective sample size’. Similarly, the imputed information score is a measure of genotype information content, which is related to the effective sample size (power) for the genetic effect being estimated [1], [5], [18]. Although computed using a different approach, the information score is analogous to the variance ratio. For example, a SNP with an imputed information score of 0.75 indicates that the imputed SNP genotypes are equivalent to a dataset with 75% of the full sample size with precisely known genotypes.

## Results

The Illumina 1 M array covers all of the SNPs on the Illumina 550 K array. We started with all SAGE subjects genotyped on the Illumina 1 M array and extracted the 545,966 SNPs that are present on the Illumina 550 K SNPs. We used these Illumina 550 K SNPs to impute to the full Illumina 1 M array. We imputed 262,864 autosomal SNPs in 2597 European Americans (EA), and 304,425 autosomal SNPs in 1264 African Americans (AA). We compared imputed SNPs to the genotyping results from the Illumina 1 M array. The remaining SNPs could not be evaluated due to the absence of those SNPs in either the Illumina 1 M array or reference panel.

The imputation results are given in Table 2. The mean IQS is lower than the mean accuracy in both EA and AA. There are cases where IQS is negative, indicating that imputation did worse than chance in assigning genotypes. In this situation, 95% of the minor allele frequencies lie between 0 and 0.058, 95% of the chance agreement rates lie between 0.78 and 1, and the imputation accuracy is below chance agreement with 95% of the values between 0.81 and 1. These are strong examples of how imputation accuracy can be misleading when “chance” contributes so strongly to the proportion of agreement.

**Table 2:** Summary of evaluation measures for European American and African American samples.

Ethnic group		European Americans	African Americans
**Evaluation Measures**	No. of imputed SNPs	260908	304425
**Imputation Accuracy**	Efficiency %	94.5	85.1
	Mean %	98.8	97.1
	Range %	0.0∼100.0	0.0∼100.0
	Inter-quartile %	98.8∼99.9	96.3∼99.5
**Imputation Quality Score (IQS)**	Mean %	90.2	78.3
	Range %	−9.1∼100	−7.9∼100
	Inter-quartile %	90.7∼99.2	68.4∼94.3

A second notable result is that the quality of imputation in AA is markedly lower than in EA. This is seen in the decreased efficiency by nearly ten percentage points, and decrease in mean IQS by nearly twelve percentage points. This is likely due to two factors. First, African Americans have more diverse haplotypes and more uncommon alleles. Second, there is non-negligible difference between African Americans and the YRI reference panel, which was clearly reflected by Eigenstrat population structure analysis [17]. Interestingly, imputation accuracy is nearly the same for EA as for AA, again highlighting how imputation accuracy can overestimate the quality of imputation.

**Figure 1:**
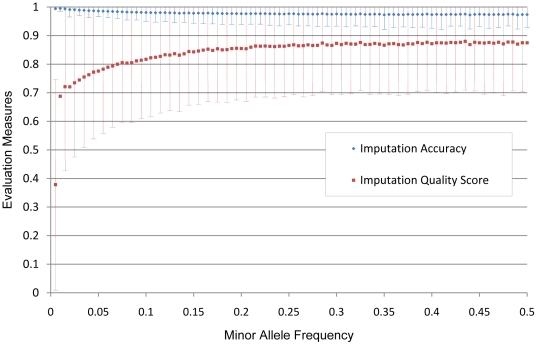
The means of IQS and imputation accuracy within each minor allele frequency interval. IQS adjusts for chance agreement. As the minor allele frequency approaches 0, the difference between IQS and imputation accuracy increases. The standard deviation is shown for every other point.

The relationship between IQS and imputation accuracy with respect to minor allele frequency is seen in Figure 1. Although imputation accuracy increases with decreased minor allele frequency, IQS drops dramatically with decreased minor allele frequency. Because it is known that low minor allele frequency decreases the quality of imputation, many studies drop SNPs with minor allele frequency less than 1%. According to this plot, this practice would still retain SNPs with an average IQS score of 88%, and would eliminate some well-imputed SNPs.

We then evaluated the effectiveness of IQS in the situation where cases and controls are genotyped on different platforms. We randomly divided the SAGE data into two subgroups labeled “cases” and “controls”. In “cases”, original genotypes were retained for SNPs on the Illumina 550 K array; and then imputation was performed to obtain the full Illumina 1 M array. In “controls”, original genotypes were retained for all SNPs on the Illumina 1 M array. This process is equivalent to combining cases genotyped by the Illumina 550 K array and controls genotyped by the Illumina 1 M array.

**Figure 2:**
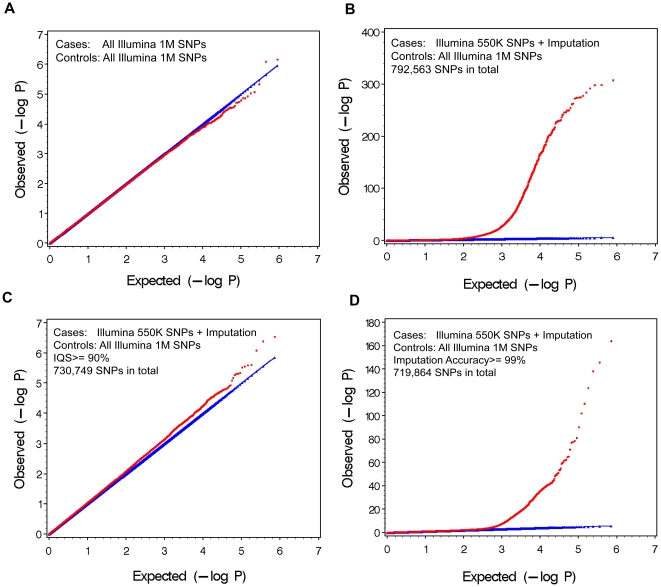
The Q-Q plots based on randomly dividing data into cases and controls. Samples were divided randomly into cases and controls. (A) All Illumina 1 M SNPs are directly genotyped indicating there is no population stratification or other non-random factors in cases and controls. (B) Cases were genotyped on the Illumina 550 K array and the remaining Illumina 1 M SNPs were imputed. (C) An IQS filter (IQS>0.9) was applied, retaining 92% of the SNPs. (D) An imputation accuracy filter (>0.99) was applied, retaining 91% of the SNPs.

We tested genetic association of all the 1 M SNPs with the cases and controls. A Quantile-Quantile Plot (Q-Q plot) is shown in Figure 2. By comparing the distribution of observed P values against the theoretical model distribution of expected P values, Q-Q plots are used in genome wide association studies to assess the inflation of false positive rates [19]. In randomized data without type I error arising from population stratification or some other artifact, the Q-Q plot should be a 45 degree line. To ensure that our random division of the data did not result in population stratification, we constructed a Q-Q plot based on the true genotypes, which was normal as expected (λ = 1.03) (Fig. 2A). However, the Q-Q plot of imputed SNPs compared to genotyped SNPs is greatly distorted (λ = 1.15), suggesting that combining imputed SNPs with genotyped SNPs without other quality control is problematic (Fig. 2B). Therefore, the observed distortion was due to imputation error and the statistically skewed SNPs (Fig. 2B) are false positives. We then filtered the imputed data by removing all SNPs with IQS≤0.9, retaining 76% of the imputed SNPs, and dramatically improving the Q-Q Plot (λ = 1.04) (Fig. 2C). The Q-Q plot remained grossly distorted even when the filter was changed to an imputation accuracy of >99%, retaining 72% of the SNPs, although λ improved to 1.05 (Fig. 2D). Although this is a very strict value for imputation accuracy, the Q-Q plot clearly shows there is significant type I error.

A more practical way of evaluating this approach is to look at the false positive rate. Specifically, although no SNPs are associated with case/control status based on the true genotypes, there were 4016 imputed SNPs that reach genome-wide significance (p<5×10^−8^). The IQS filter >0.9 eliminated all the false positive SNPs, but the imputation accuracy filter >0.99 still retained 759 false-positive SNPs. Based on these results, IQS is better for discriminating between well-imputed SNPs and poorly-imputed SNPs.

Although IQS can serve as an effective filter to minimize the use of poorly-imputed SNPs, the computation of IQS requires a sample that was both imputed and genotyped for the SNPs of interest. This is impractical in most situations. A secondary goal of this paper is to determine whether there are ways to evaluate imputation quality without knowing the true genotypes.

The two common methods for filtering imputed data are to combine a minor allele frequency threshold with either the imputed information score >0.3∼0.5 (PROPER_INFO in SNPTEST) [10], [18], [20], [21], [22] or the variance ratio >0.3 (rsq_hat in MACH) [7], [10], [20], [21], [22], [23], [24], [25]. We calculated these two statistics for our data and compared these filters to IQS (Table 3). After filtering by these statistics, the type I error inflation decreases. In the AA sample, IQS also acts as an effective filter and can be cautiously approximated by a combination of MAF and either the imputed information score or the variance ratio (Table S1). Unfortunately, even in the most conservative situation, over three thousand false positives remain. Therefore this is an ineffective approach for filtering poorly-imputed SNPs.

**Table 3:** Comparison of empirical evaluations of imputation quality to IQS in European Americans.

		Minor Allele frequency	
False positives n (retained %)	>0.01	>0.05	>0.10
IQS >0.9	0 (89.47%)	0 (83.90%)	0 (72.92%)
No filter	3120 (96.63%)	2331 (89.48%)	1775 (77.47%)
Proper_info >0.5	3093 (96.62%)	2329 (89.48%)	1775 (77.47%)
Proper_info >0.7	2726 (96.32%)	2080 (89.28%)	1571 (77.31%)
Proper_info >0.9	1392 (94.16%)	1032 (87.67%)	805 (76.06%)
Variance Ratio >0.3	1869 (96.22%)	1526 (89.27%)	1234 (77.33%)
Variance Ratio >0.5	1226 (95.65%)	928 (88.89%)	770 (77.04%)
Variance Ratio >0.7	789 (94.57%)	514 (88.12%)	390 (76.47%)
Variance Ratio >0.9	498 (90.40%)	253 (85.00%)	153 (74.14%)
MAF difference <0.01	267 (22.89%)	120 (19.63%)	76 (15.60%)
MAF difference <0.1	2516 (95.11%)	1739 (87.97%)	1191 (75.94%)
MAF difference <0.2	2952 (96.57%)	2168 (89.42%)	1615 (77.38%)

The sample is based on 2,597 European Americans that were randomized to cases and controls. Cases used genotypes from the Illumina 550 K platform and were imputed to the 1 M platform and controls were genotyped on the 1 M platform. Genome-wide significance is set as p<5×10^−8^. There were 792,563 SNPs available. False positives refer to the absolute number of SNPs that reached genome-wide significance despite the filter. The retained percentage is the proportion of SNPs that passed the filter.

Filtering on MAF differences between the Hapmap and the study genotypes is another possible approach to control false positives. In Table 3 and Supplementary Table S1, we provided results filtered by MAF difference at 0.01, 0.1 and 0.2 for European Americans and African Americans, respectively. Filtering by MAF difference of 0.01 resulted in a reduction of false positives, but retained less than 25% of the SNPs. In contrast, filtering with a MAF difference of 0.1 or 0.2 retained many false positives.

**Figure 3:**
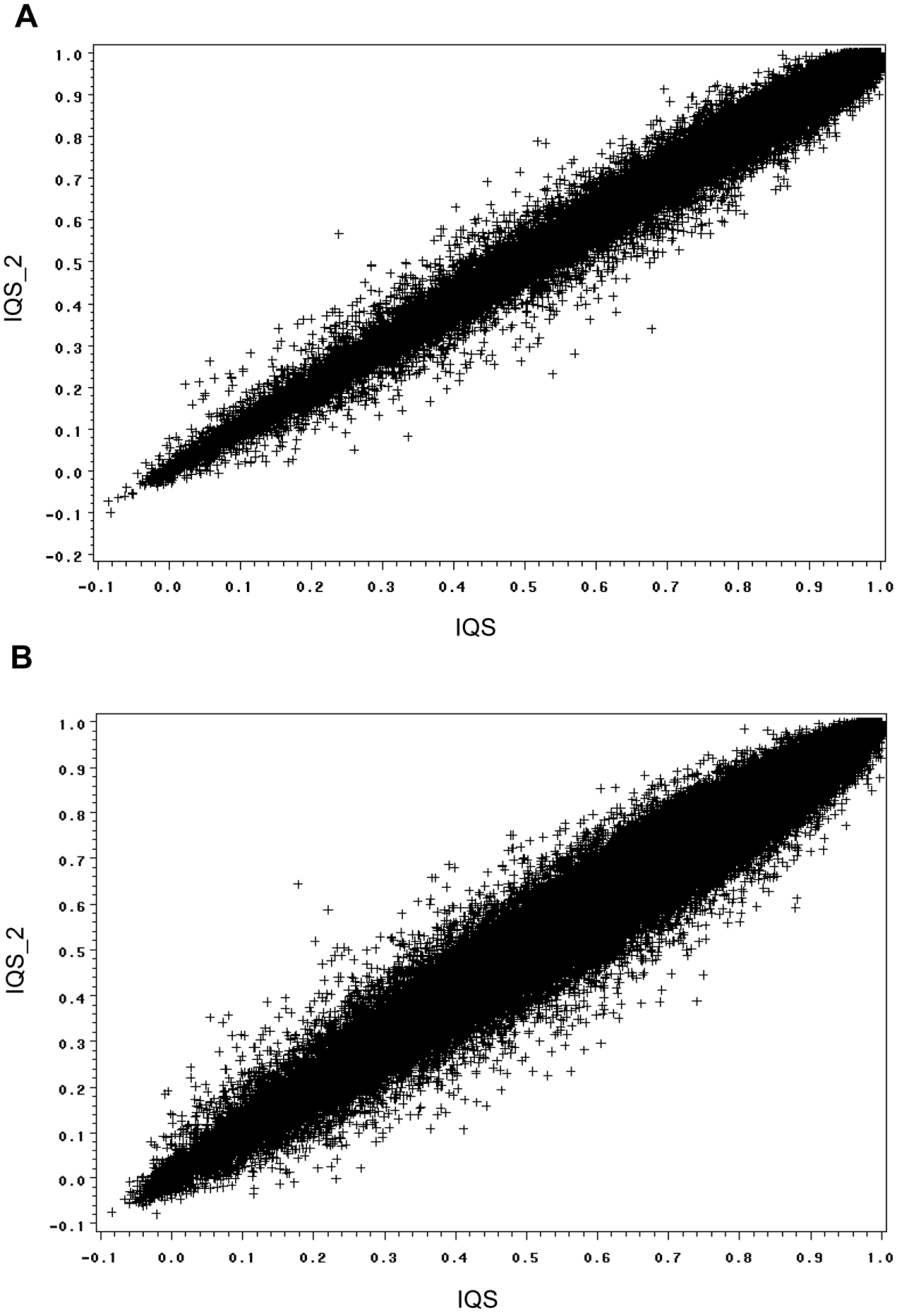
Evaluation of the robustness of IQS score. European Americans (A) and African Americans(B) datasets were split in half and Illumina 550 K SNPs were imputed to Illumina 1 M SNPs. IQS score for the two halves of the data were plotted against each other. SNPs with minor allele frequency less than 0.01 were excluded to avoid zero in the denominator.

A second method for using IQS without directly genotyping would be to develop a database of common imputations in common populations that records IQS scores for each SNP. To test the practicality of this approach, we randomly divided the data into two groups and tested the robustness of the IQS score for the SNPs imputed from the Illumina 550 K array to the Illumina 1 M array in both EA and AA. Because small changes in the denominator of IQS (1-P_c_) will dramatically affect the value of the statistic when MAF is small, we included only SNPs with MAF>0.01. Figure 3 plots the IQS scores in both populations. The correlation in EA is 0.99519 and the correlation in AA is 0.99020, indicating that IQS is robust for the same imputation in a relatively homogeneous population.

**Figure 4:**
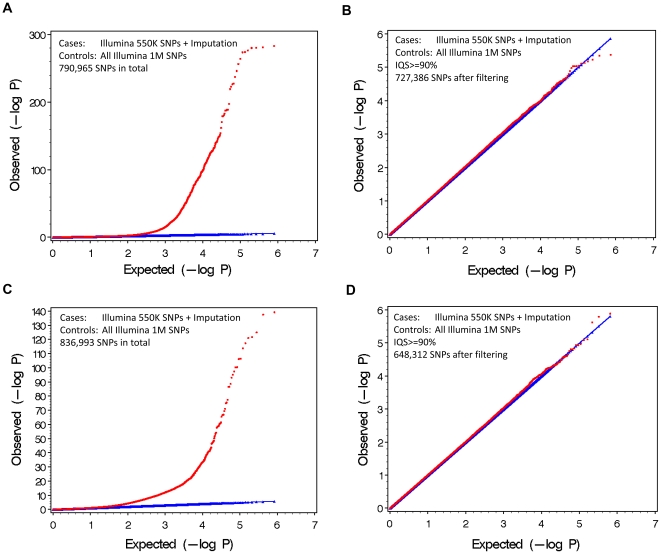
A database of IQS can be used to filter poorly-imputed SNPs. The set of hard-to-impute SNPs compiled from one dataset can be used to filter the imputed data in another dataset. (A) Cases were European Americans genotyped on the Illumina 550 K array and the remaining Illumina 1 M SNPs were imputed. Controls were European Americans genotyped on the Illumina 1 M array. The QQ plot was shown for the 790,965 available SNPs. (B) An IQS filter (IQS>0.9) was applied, retaining 92% of the SNPs. IQS was calculated from an independent dataset. (C) A similar QQ plot for African Americans. Cases were genotyped on the Illumina 550 K array and the remaining Illumina 1 M SNPs were imputed. Controls were genotyped on the Illumina 1 M array. The QQ plot was shown for the 836,993 available SNPs. (D) An IQS filter (IQS>0.9) was applied, retaining 78% of the SNPs. IQS was calculated from an independent dataset.

We further tested whether the set of hard-to-impute SNPs compiled from the first group can be used to filter the imputed data in the second group. We applied a similar procedure as in Figure 1. We randomly divided the second group into cases and controls. Cases were genotyped on the Illumina 550 K array and the remaining Illumina 1 M SNPs were imputed. Controls were genotyped on the Illumina 1 M array. Figure 4 shows that the QQ plot can be adjusted to normal by IQS calculated from the first group. This implies that the development of a database of IQS scores for standard imputations would allow researchers to use data genotyped on different platforms and filter out potential false positives.

In order to confirm these results in a different dataset, we replicated the study in European American subjects genotyped on two different platforms, Affymetrix 5.0 array and Illumina 550 K array. All subjects were controls from the National Institute of Mental Health Center for Collaborative Genetic Studies on Mental Disorders. We randomly divided about 400 individuals into two subgroups labeled “cases” and “controls” in a similar manner as above. “Cases” were genotyped by the Affymetrix 5.0 array and “controls” were genotyped by the Illumina 550 K array. In the replication, we also expanded our investigation to include those SNPs that were not genotyped in either array, but were imputed from their respective arrays. In fact, we had genotype data from both platforms. No genome wide significant SNPs were found. Therefore, if there were any significant SNPs in this simulation, they should be false positives. The result was similar with inflation of Type I error that is effectively filtered by IQS, whereas filtering by MAF and either the imputed information score or the variance ratio continue to have many false positive values (Table S2).

## Discussion

There are two situations in which imputation is avoided[18]: (1) SNPs with low minor allele frequency and (2) cases and controls genotyped on different platforms. The statistics previously used for measuring the accuracy of imputation are inadequate for evaluating the quality of imputation due to their dependence on marginal SNP frequency. Specifically, imputation accuracy, a measure of the concordance rate between the imputed and observed genotypes for each SNP, dramatically over-estimates reliability when minor allele frequencies are low and does not address the inflation of false positive rates arising from imputation error due to random agreement. We developed IQS to more precisely estimate imputation error, effectively filtering imputation error in these two problematic situations. We showed that IQS is a more appropriate measure to evaluate imputation reliability because it adjusts for “chance” agreement, and filtering by IQS eliminates the inflation of the false positive rate arising from imputation error.

It is important to note that the traditional genome inflation factor λ is not an ideal indicator of potential problems related to imputation quality. In our studies, we noticed that λ is not dramatically different from 1, in contrast to the extent that the Q-Q plot is distorted (Fig. 2B D). The reason is that λ reflects systematic inflation on all SNPs while the distortion of the Q-Q plot in our studies is due to a small number of poorly-imputed SNPs. However, problems with this limited number of SNPs (less than 0.5% of total SNPs) can be dramatic and lead to pronounced false positive P values that exceed genome wide significance.

We also would like to emphasize that we are dealing with the extreme situation when cases and controls are genotyped on different platforms. The elevated false positive rates are not explicitly reported in the literature, as most groups do not have this problem because of the study design. But many groups have noticed it. In a recent paper by de Bakker[18], the author noted “the dangers of combining cases genotyped on one platform and controls genotyped on another” (Page 124). In the GENEVA consortium, there is a consensus that genotypes imputed from one array should not be combined with imputed genotypes from another array.

The reasons for the false positives are very complicated. Among the 4016 genome wide significant SNPs, most of them have low R square with other available SNPs. It is difficult to correctly assign their values based on related haplotypes, and they therefore tend to receive the allele frequency from the reference panel.

Filtering by the difference between the reference and the estimated minor allele frequency can effectively remove some genome wide significant SNPs. Of the 4016 genome wide falsely-significant SNPs, 3120 (77.7%) SNPs are removed by removing those SNPs whose minor allele frequency difference is greater than 0.01.However, there are still 832 (21% of the 4016 SNPs) that have passed the filter. Most of the 832 remaining SNPs share one character: they tend to have very low minor allele frequency (MAF median  = 0.00096). Imputation tends to over-assign the major genotype to the imputed SNPs, resulting in different allele frequency and therefore inflating the P value. However, to filter by MAF difference at 0.01 is not an acceptable option. Most SNPs are correctly predicted even if the minor allele frequency is different. When we tried to remove all SNPs whose minor allele frequency difference was greater than 0.01, 583,456 of the total 788,944 available SNPs (74%) were removed. Most of these SNPs were correctly predicted even if minor allele frequency was different. This is because imputation does not assign predicted genotype based on minor allele frequency, but rather on haplotype modeling.

The typical methods for filtering poorly-imputed SNPs are using either the variance ratio or the imputed information score combined with minor allele frequency. Imputation quality is especially important in a study that combines genotypes from different platforms. Therefore, we increased our thresholds for variance ratio and the imputed information score in Table 3, and in Supplementary Tables S1 and S2. But these measures were ineffective in this extreme situation. However, IQS may be used as an effective filter to combine data genotyped on different platforms.

Because IQS requires direct genotyping for evaluation, it is not a practical statistic for directly evaluating imputation in the case where imputation is used to screen for associations as a proxy for genotyping. However, IQS was shown to be a robust measure of imputation for specific imputations (from one standard platform to another) and within a broad population (tested in both EA and AA).

Generally speaking, different populations have different linkage disequilibrium structures and different allele frequencies that lead to different IQS values. A mixture of different populations will make the IQS sensitive to the ratio of population mixture. Therefore, as in general association studies, a mixture of different populations should be avoided. However, African Americans have a unique and relatively stable genetic structure. The IQS score from African Americans is stable in our study and is useful to filter out poorly imputed SNPs.

Based on this theory, a database can be constructed and used to filter future imputations and to avoid false positive associations. In order to advance the development of this database, we have posted IQS scores for imputation from Illumina 550 K to Illumina 1 M for CEPH on the website of the NIMH Center (http://www.nimhgenetics.org/). We envision this as a dynamic database to be updated when new datasets include subjects genotyped on multiple platforms. We will further provide IQS scores for various array combinations when the genotype data of 6,000 controls typed on both the Affymetrix 6.0 and Illumina 1 M array are available in the near future [26]. The future database will include IQS scores for the following imputations: (1) from Affymetrix 6.0 to Illumina 1 M, (2) from Illumina 1 M to Affymetrix 6.0, (3) from Illumina 300 K to Affymetrix 6.0 plus Illumina 1 M, (4) from Illumina 550 K to Affymetrix 6.0 plus Illumina 1 M, and (5) from Affymetrix 5.0 to Affymetrix 6.0 plus Illumina 1 M. Although genotyping will be ultimately required to confirm associations, using IQS as a filter will decrease the amount of false positive findings that arise, making follow up of positive associations practical.

As genome wide association studies move toward rare variants, over-estimation of the quality of imputation due to chance concordance of uncommon alleles will be more common. In addition, imputation will and should be used to analyze increasingly complex data structures. IQS can be used as an accurate evaluation of imputation quality enabling researchers to examine low allele frequencies and complex data structures.

## Supporting Information

Table S1 Comparison of empirical evaluations of imputation quality to IQS in African Americans. The sample is based on 1,264 African Americans that were randomized to cases and controls. Cases used genotypes from the Illumina 550K platform and were imputed to the 1M platform and controls were genotyped on the 1 M platform. Genome-wide significance is set as p<5E-8. There were 837,001 SNPs available. False positives refer to the absolute number of SNPs that reached genome-wide significance despite the filter. The retained percentage is the proportion of SNPs that passed the filter.
(0.04 MB DOC)

Table S2 Comparison of empirical evaluations of imputation quality to IQS when combining Affymetrix 5.0 and Illumina 550 K SNPs. The sample is based on 418 healthy European Americans from the NIMH Repository. Cases were genotyped on the Affymetrix 5.0 platform and were imputed to the Illumina 550 platform and controls were genotyped on the Illumina 550 platform and imputed to the Affymetrix 5.0 platform. Genome-wide significance is set as p<5E-8. There were 2,553,465 SNPs available (including Hapmap SNPs). False positives refer to the absolute number of SNPs that reached genome-wide significance despite the filter. The retained percentage is the proportion of SNPs that passed the filter.
(0.04 MB DOC)
