# Supplementary material for: A New Statistic to Evaluate Imputation Reliability
Source: PLoS One. 2010 Mar 15;5(3):e9697. doi: 10.1371/journal.pone.0009697 (PMC2837741; doi:10.1371/journal.pone.0009697)
Supplement: Table S1 — Comparison of empirical evaluations of imputation quality to IQS in African Americans. The sample is based on 1,264 African Americans that were randomized to cases and controls. Cases used genotypes from the Illumina 550K platform and were imputed to the 1M platform and controls were genotyped on the 1 M platform. Genome-wide significance is set as p<5E-8. There were 837,001 SNPs available. False positives refer to the absolute number of SNPs that reached genome-wide significance despite the filter. The retained percentage is the proportion of SNPs that passed the filter. (0.04 MB DOC) [file pone.0009697.s001.doc]

**Table S1. Comparison of empirical evaluations of imputation quality to IQS in African Americans.**

|  |  | | Minor Allele frequency |  |
| --- | --- | --- | --- | --- |
| False positives n (retained %) | >0.01 | >0.05 | | >0.10 |
| IQS > 0.9 | 1 (76.82%) | 1 (70.69%) | | 1 (61.99%) |
| No filter | 8634 (99.35%) | 7671 (90.77%) | | 5537 (77.72%) |
| Proper_info >0.5 | 8620 (99.35%) | 7665 (90.77%) | | 5537 (77.72%)) |
| Proper_info >0.7 | 8087 (98.83%) | 7254 (90.37%) | | 5205 (77.43%) |
| Proper_info >0.9 | 3715 (90.72%) | 3243 (83.52%) | | 2058 (72.45%) |
| Variance Ratio >0.3 | 4728 (98.32%) | 4185 (90.04%) | | 3530 (77.30%) |
| Variance Ratio >0.5 | 3059 (96.68%) | 2524 (88.66%) | | 1976 (76.33%) |
| Variance Ratio >0.7 | 2092 (93.09%) | 1570 (85,49%) | | 1054 (73.97%) |
| Variance Ratio >0.9 | 1518 (81.03%) | 1020 (74.97%) | | 557 (65.59%) |
| MAF difference <0.01 | 348 (14.05%) | 241 (12.44%) | | 159 (9.97%) |
| MAF difference <0.1 | 6288 (90.53%) | 5331 (81.89%) | | 3227 (68.81%) |
| MAF difference <0.2 | 8461 (99.22%) | 7495 (90.57%) | | 5363 (77.46%) |

The sample is based on 1,264 African Americans that were randomized to cases and controls. Cases used genotypes from the Illumina 550K platform and were imputed to the 1M platform and controls were genotyped on the 1M platform. Genome-wide significance is set as p<5E-8. There were 837,001 SNPs available. False positives refer to the absolute number of SNPs that reached genome-wide significance despite the filter. The retained percentage is the proportion of SNPs that passed the filter.
