# Supplementary material for: A New Statistic to Evaluate Imputation Reliability
Source: PLoS One. 2010 Mar 15;5(3):e9697. doi: 10.1371/journal.pone.0009697 (PMC2837741; doi:10.1371/journal.pone.0009697)
Supplement: Table S2 — Comparison of empirical evaluations of imputation quality to IQS when combining Affymetrix 5.0 and Illumina 550 K SNPs. The sample is based on 418 healthy European Americans from the NIMH Repository. Cases were genotyped on the Affymetrix 5.0 platform and were imputed to the Illumina 550 platform and controls were genotyped on the Illumina 550 platform and imputed to the Affymetrix 5.0 platform. Genome-wide significance is set as p<5E-8. There were 2,553,465 SNPs available (including Hapmap SNPs). False positives refer to the absolute number of SNPs that reached genome-wide significance despite the filter. The retained percentage is the proportion of SNPs that passed the filter. (0.04 MB DOC) [file pone.0009697.s002.doc]

**Table S2. Comparison of empirical evaluations of imputation quality to IQS when combining Affymetrix 5.0 and Illumina 550K SNPs.**

|  |  | | Minor Allele frequency |  |
| --- | --- | --- | --- | --- |
| False positives n (Retained %) | >0.01 | >0.05 | | >0.10 |
| No filter | 2047 (97.09%) | 1536 (85.55%) | | 1107 (72.97%) |
| IQS > 0.5 | 63 (87.5%) | 63 (78.48%) | | 60 (67.30%) |
| IQS > 0.7 | 2 (80.52%) | 2 (72.78%) | | 2 (62.64%) |
| IQS > 0.9 | 0 (62.99%) | 0 (57.62%) | | 0 (49.87%) |
| Proper_info >0.5 | 1550 (94.43%) | 1280 (84.12%) | | 979 (71.89%) |
| Proper_info >0.7 | 1300 (90.46%) | 1063 (81.23%) | | 818 (69.74%) |
| Proper_info >0.9 | 635 (77.49%) | 479 (70.43%) | | 338 (60.98%) |
| Variance Ratio >0.3 | 1657 (95.40%) | 1285 (84.34%) | | 937 (72.05%) |
| Variance Ratio >0.5 | 1138 (92.43%) | 904 (82.12%) | | 673 (70.33%) |
| Variance Ratio >0.7 | 729 (87.17%) | 562 (77.90%) | | 434 (66.93%) |
| Variance Ratio >0.9 | 427 (73.31%) | 318 (65.58%) | | 257 (56.39%) |

The sample is based on 418 healthy European Americans from the NIMH Repository. Cases were genotyped on the Affymetrix 5.0 platform and were imputed to the Illumina 550 platform and controls were genotyped on the Illumina 550 platform and imputed to the Affymetrix 5.0 platform. Genome-wide significance is set as p<5E-8. There were 2,553,465 SNPs available (including Hapmap SNPs). False positives refer to the absolute number of SNPs that reached genome-wide significance despite the filter. The retained percentage is the proportion of SNPs that passed the filter.
